# Supplementary material for: Crawling and Gliding: A Computational Model for Shape-Driven Cell Migration
Source: PLoS Comput Biol. 2015 Oct 21;11(10):e1004280. doi: 10.1371/journal.pcbi.1004280 (PMC4619082; doi:10.1371/journal.pcbi.1004280)
Supplement: S1 Code — (ZIP) [file pcbi.1004280.s012.zip › release/tst/doc/html/classes.html]

Tissue Simulation Toolkit: Class Index


Tissue Simulation Toolkit
 0.1.4.1


- Main Page
- Namespaces
- Classes
- Files

- Class List
- Class Hierarchy
- Class Members

Class Index

C | D | G | I | L | P | Q | X

|  |  |  |  |  |
| --- | --- | --- | --- | --- |
| C | Dish | P | X | l |
|  | G |  |  |  |
| Cell |  | Parameter | X11Graphics | li |
| CellularPotts | Graphics | PDE | c |  |
| D | I | Point |  |  |
|  |  | Q | co |  |
| Dir | Info |  |  |  |
|  |  | QtGraphics |  |  |
|  |  |  |  |  |

C | D | G | I | L | P | Q | X


---

Generated on Thu Aug 14 2014 22:04:01 for Tissue Simulation Toolkit by  

 1.8.6
